# Citrate trafficking supports rewiring of mitochondrial metabolism via RTG signaling in yeast osmoadaptation

**DOI:** 10.1042/BCJ20253414

**Published:** 2025-12-17

**Authors:** Angela Primavera, Luna Laera, Alessandra Castegna, Pasquale Scarcia, Luigi Palmieri, Maria Antonietta Di Noia, Nicoletta Guaragnella

**Affiliations:** 1 Department of Biosciences, Biotechnology and Environment, University of Bari "Aldo Moro", Bari, Italy; 2 Institute of Biomembranes, Bioenergetics and Molecular Biotechnologies, National Research Council, Bari, Italy

**Keywords:** citrate, mitochondrial carriers, osmoadaptation, RTG pathway, yeast, *YHM2*

## Abstract

Inter-organellar cross-talk is an important component of cellular stress response enabling adaptation and survival. We have demonstrated the activation of RTG retrograde signaling to sustain the peroxisomesmitochondria– nucleus axis in a model of osmostressed *Saccharomyces cerevisiae* yeast cells. In this work, we aimed to gain insight into the molecular mechanisms regulating the communication between these organelles upon NaCl treatment. A metabolomic analysis revealed that the homeostasis of citrate is a pivotal factor in the osmoadaptive response. Gene expression analysis and citrate synthase activity showed that the synthesis of citrate mainly derives from peroxisomes, as indicated by the up-regulation of *CIT2*, and not *CIT1* and *CIT3*, under the control of the RTG pathway. Furthermore, the involvement of the mitochondrial citrate transporter, encoded by *YHM2*, in the osmoadaptive response, as judged by gene and protein expression analysis together with growth assay, is demonstrated. In the absence of *YHM2*, alternative pathways relying on *ODC2* and *ACO1* are activated, indicating possible compensatory mechanisms for osmoadaptation. We propose a model in which peroxisome-derived citrate is converted to cytosolic 2-oxoglutarate to replenish TCA cycle and promote its rewiring. This work reveals a new layer of metabolic co-ordination among organelles and identifies citrate shuttling as a crucial adaptive mechanism to osmotic stress.

## Introduction

Cells experience a variety of stressors throughout their life cycle, ranging from oxidative and osmotic stress to nutrient deprivation and temperature fluctuations. To cope with these challenges, cells must activate adaptive strategies, such as stress response signaling pathways, autophagy, and organelle remodeling, or else succumb to regulated cell death pathways such as apoptosis or necrosis [1]. The balance between survival and death is governed by a dynamic and complex network of transcriptional and molecular interactions, both within individual pathways and between different subcellular compartments. In this context, organelle cross-talk has emerged as a critical aspect of cellular stress response. Such interactions enable rapid co-ordination of metabolic flux, reactive oxygen species (ROS) handling, calcium signaling, and the distribution of lipids and proteins necessary for adaptation [2–4]. Mitochondria are now widely recognized as key signaling centers within the cell, sensing intracellular and extracellular perturbations and relaying signals to other organelles [5,6]. They continuously monitor fluctuations in both intra- and extracellular conditions and co-ordinate responses by communicating with other subcellular structures, including nucleus, endoplasmic reticulum, and peroxisomes. The communication between mitochondria and nucleus is termed retrograde signaling and appears evolutionary conserved from yeast to humans [7]. Its main function is to restore metabolic homeostasis, especially, but not exclusively, in the case of mitochondrial dysfunction. The mechanism of mitochondrial retrograde signaling has been described in *Saccharomyces cerevisiae* and relies on RTG genes, with Rtg2 promoting the nuclear translocation of the Rtg1–Rtg3 transcription factor complex to activate compensatory metabolic pathways for cell adaptation to mitochondrial impairment [8]. The contribution of mitochondrial retrograde signaling to yeast stress response and cellular adaptation has been described in several other cases, including acid stress, endoplasmic reticulum stress, as well as oxidative and osmotic stress [9–14]. In particular, our group demonstrated that the RTG pathway supports long-term adaptation to osmotic stress downstream of Hog1, the main kinase of HOG pathway [15]. Furthermore, we demonstrated a functional interaction between *RTG2* and *HAP4*, a key regulator of mitochondrial biogenesis and respiratory function: deletion of *HAP4* accelerates the kinetics of osmoadaptation and is characterized by the up-regulation of the first three genes of the TCA cycle, *CIT1*, *ACO1,* and *IDH1*. These effects are largely *RTG*-dependent and occur despite reduced respiratory function, suggesting that RTG signaling is particularly relevant under conditions of compromised mitochondrial activity [16]. Peroxisomal activity has been recognized as crucial upon salt stress [17], and an inter-organellar cross-talk between mitochondria, nucleus, and peroxisomes has been hypothesized to sustain mitochondrial function under osmotic stress conditions [16].

Recent studies in yeast and in mammalian cells revealed that peroxisomes interact functionally and physically with mitochondria to support metabolic co-ordination and redox activities, especially under stress conditions. Evidence suggests that peroxisomes may form transient contact sites with mitochondria, potentially involving components of tethering structures, thereby contributing to inter-organelle communication and metabolic integration [18–23]. These interactions are crucial for the dynamic adaptation of cells to changing metabolic demands and stressors, further emphasizing the importance of mitochondrial connectivity with multiple organelles, including peroxisomes [24]. Although this aspect is of critical importance, the molecular mechanisms and metabolic signals involved in organelles communication under stress conditions remain poorly understood.

Here, we investigated how metabolic cross-talk between mitochondria, nucleus, and peroxisomes contributes to osmoadaptation in *Saccharomyces cerevisiae*. Our findings revealed that the levels of citrate are determinant for metabolic rewiring and stress adaptation. In this context, the involvement of *YHM2*, encoding the mitochondrial citrate/2-oxoglutarate (2-OG) transporter [25], is also shown. This work provides a dynamic model in which mitochondria act as sensors and transmitters of stress signals and establish an active co-operation with nucleus and peroxisomes. Interestingly, our results uncover a novel layer of retrograde regulation, where mitochondrial transporters [26,27] actively contribute to inter-organellar communication and metabolic rewiring required for adaptation to hyperosmotic conditions.

## Results

### Citrate levels are increased upon NaCl stress

**Figure 1:**
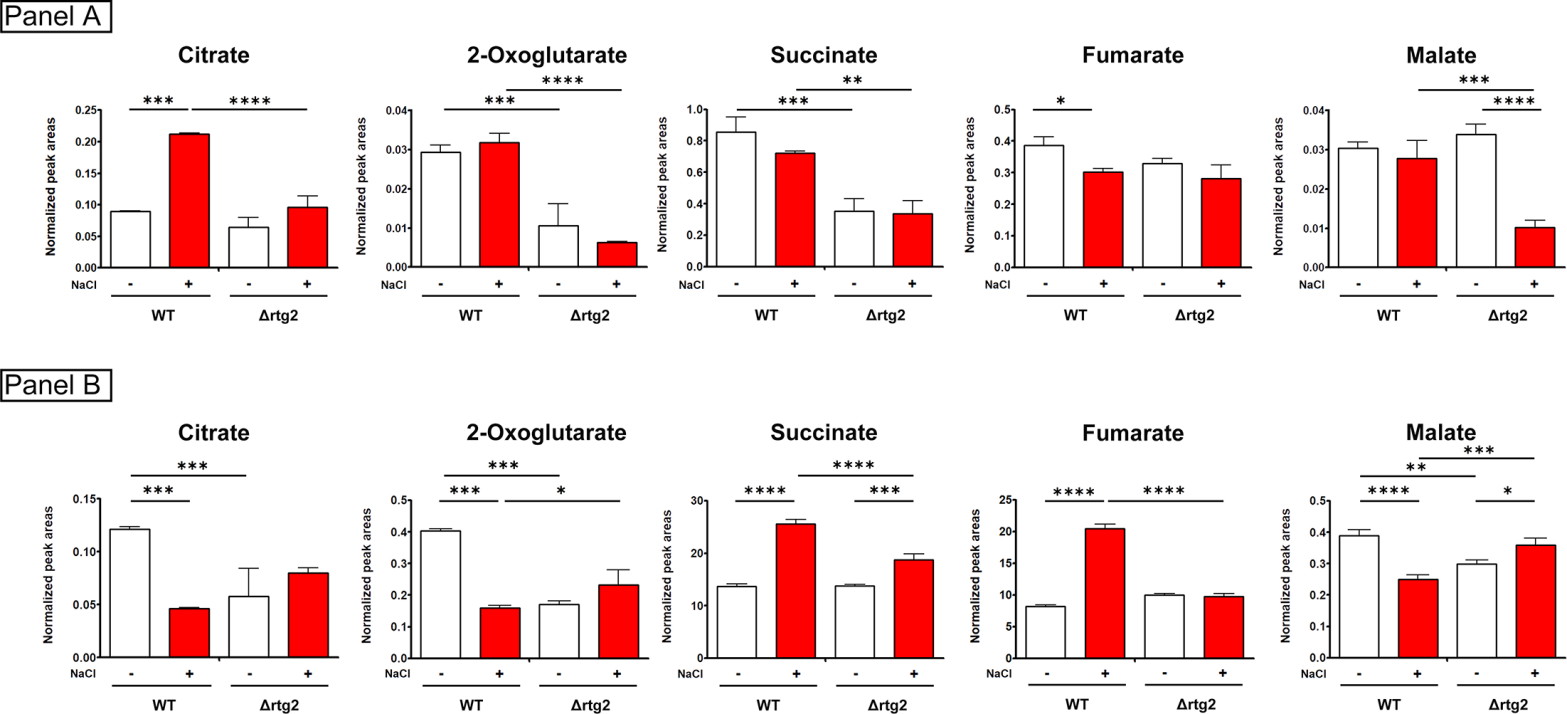
TCA cycle metabolite levels in wildtype and Δrtg2 cells upon osmostress. Intracellular (Panel A) and extracellular levels (Panel B) of citrate, 2-oxoglutarate, succinate, fumarate, and malate in WT and Δrtg2 cells after 8 h of growth in YPD in the absence (white bars) and in the presence (red bars) of 0.8 M NaCl. Data represent SD from *N* ≥ 3 biological replicates. One-way ANOVA test, *P<0.05.

In our previous work, we have hypothesized that citrate might play a major role in *RTG*-dependent osmoadaptive response of yeast cells [16]. To gain insight into this aspect, a quantification of TCA cycle metabolites was performed on wildtype (WT) and Δrtg2 cells in the absence and in the presence of NaCl by LC-MS (Figure 1). As reported in Figure 1 panel A an increase of citrate (2-fold) and a slight decrease of succinate and fumarate could be observed in stressed WT compared with control cells. Accordingly, citrate and 2-oxoglutarate decreased in the medium of NaCl-treated WT cells (Figure 1 panel B), suggesting enhanced intracellular retention and recycling of these metabolites. An increase in extracellular succinate and fumarate was observed in NaCl-treated WT cells (Figure 1 panel B), which may suggest a selective export of these TCA intermediates as part of a stress-adaptive response. Δrtg2 mutant cells exhibited a distinct metabolic profile; aside from a reduction in intracellular malate upon NaCl stress, no significant changes in the levels of other metabolites were detected, either intracellularly or in the culture medium. This specific decrease in malate may reflect reduced production via the glyoxylate cycle, likely due to impaired *CIT2* induction. By comparing WT and Δrtg2 stressed cells, it is evident that most of the metabolites are significantly decreased in the mutant, further supporting the role of *RTG2* in maintaining metabolic homeostasis during osmotic stress.

### Citrate synthesis mainly depends on the peroxisomal isoform of citrate synthase

**Figure 2:**
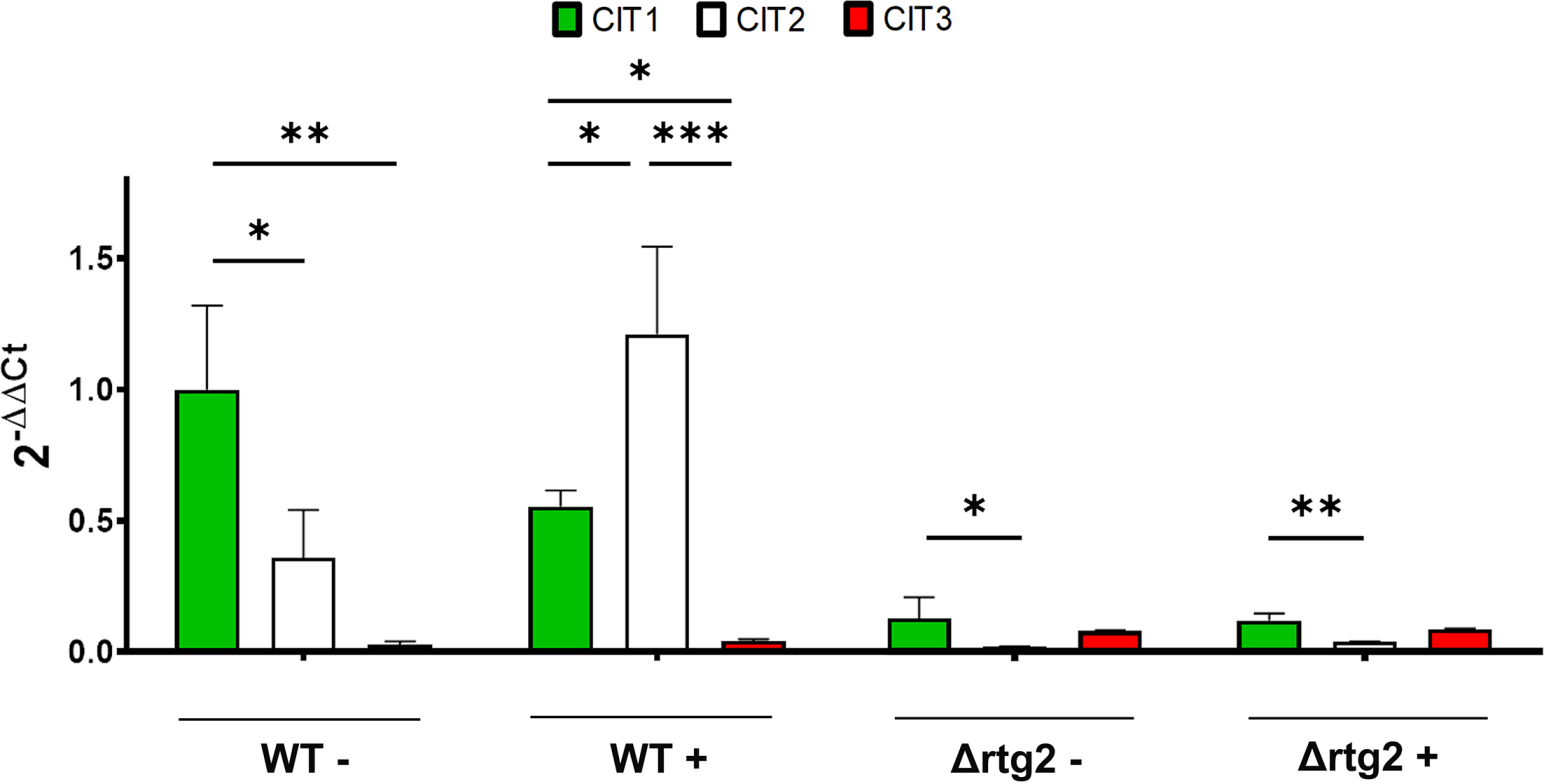
*CIT1*, *CIT2*, and *CIT3* expression under high-osmotic environment. Wildtype (WT) and Δrtg2 cells grown overnight in YPD medium were diluted to 0.1 OD600 in fresh liquid YPD with or without 0.8 M NaCl. After 5 h, cells were collected for RNA extraction, and mRNA levels were measured by quantitative PCR. Relative amounts of *CIT1, CIT2,* and *CIT3* mRNA were calculated according to WT untreated *CIT1* levels by comparative method (2^−ΔΔCt^). Values are mean + SD of three independent experiments. One-way ANOVA, Tukey’s (*post hoc*) test; ***P<0.001.

Having identified citrate as a key metabolite of the osmoadaptive response, the contribution of peroxisomes and mitochondria to its synthesis was evaluated by assessing the expression of the three citrate synthase isoforms, *CIT1*, *CIT2,* and *CIT3*, in WT and mutant cells with or without salt stress (Figure 2). The results obtained confirmed that, in the presence of NaCl, *CIT1* and *CIT2* were down- (two-fold) and up-regulated (four-fold), respectively, in WT cells (Figure 2). No significant changes were observed for the second mitochondrial isoform *CIT3* (Figure 2). However, it should be noted that the *CIT3* gene, encoding for the other mitochondrial isoform [28], is always expressed at much lower levels than the other two isoforms. In the mutant lacking *RTG2*, all three genes showed a very low level of expression independently of stress conditions.

**Figure 3:**
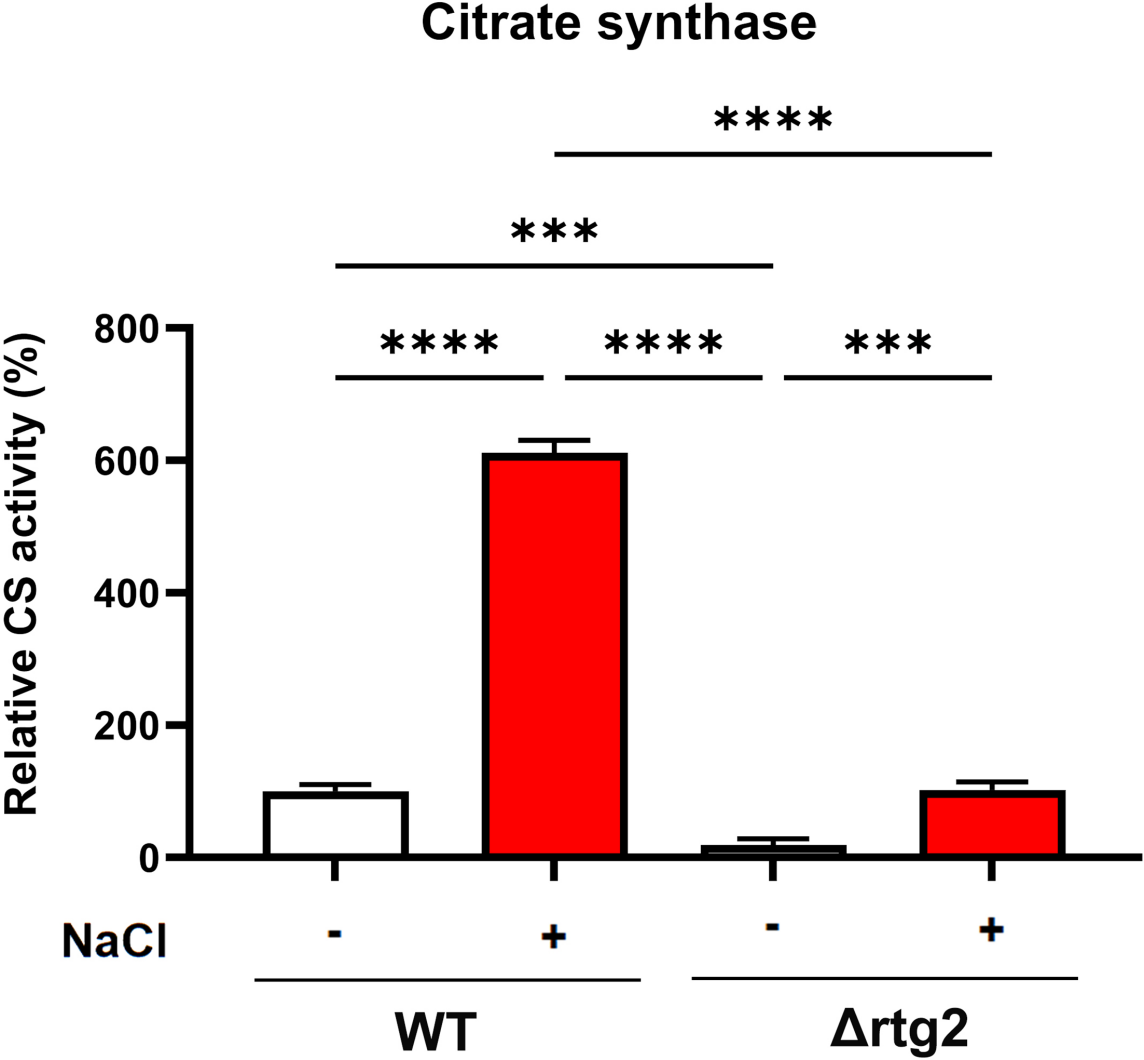
Citrate synthase activity upon salt stress. Yeast cells were grown in YPD for 8 h in the presence (red bars) or absence (white bars) of 0.8 M NaCl. The cells were processed and the specific citrate synthase enzyme activity was measured as described in materials and methods. Citrate synthase activity was measured in duplicate in three independent cultures and is expressed as a percentage of untreated WT.

In addition to transcriptional analysis, citrate synthase enzymatic activity was measured to evaluate the overall cellular catalytic response to stress conditions. As reported in Figure 3, the total activity of citrate synthase was significantly reduced in untreated Δrtg2 compared with WT cells; in the presence of NaCl, an increase in the activity could be observed in both WT and mutant cells when compared with WT untreated cells, showing an increased citrate synthase activity once. Overall, these data indicate that citrate synthesis is a key process in the osmoadaptive response of yeast cells and mainly derives from peroxisomes under the control of the RTG pathway.

### Yhm2 supports osmoadaptation by mediating citrate shuttling

**Figure 4:**
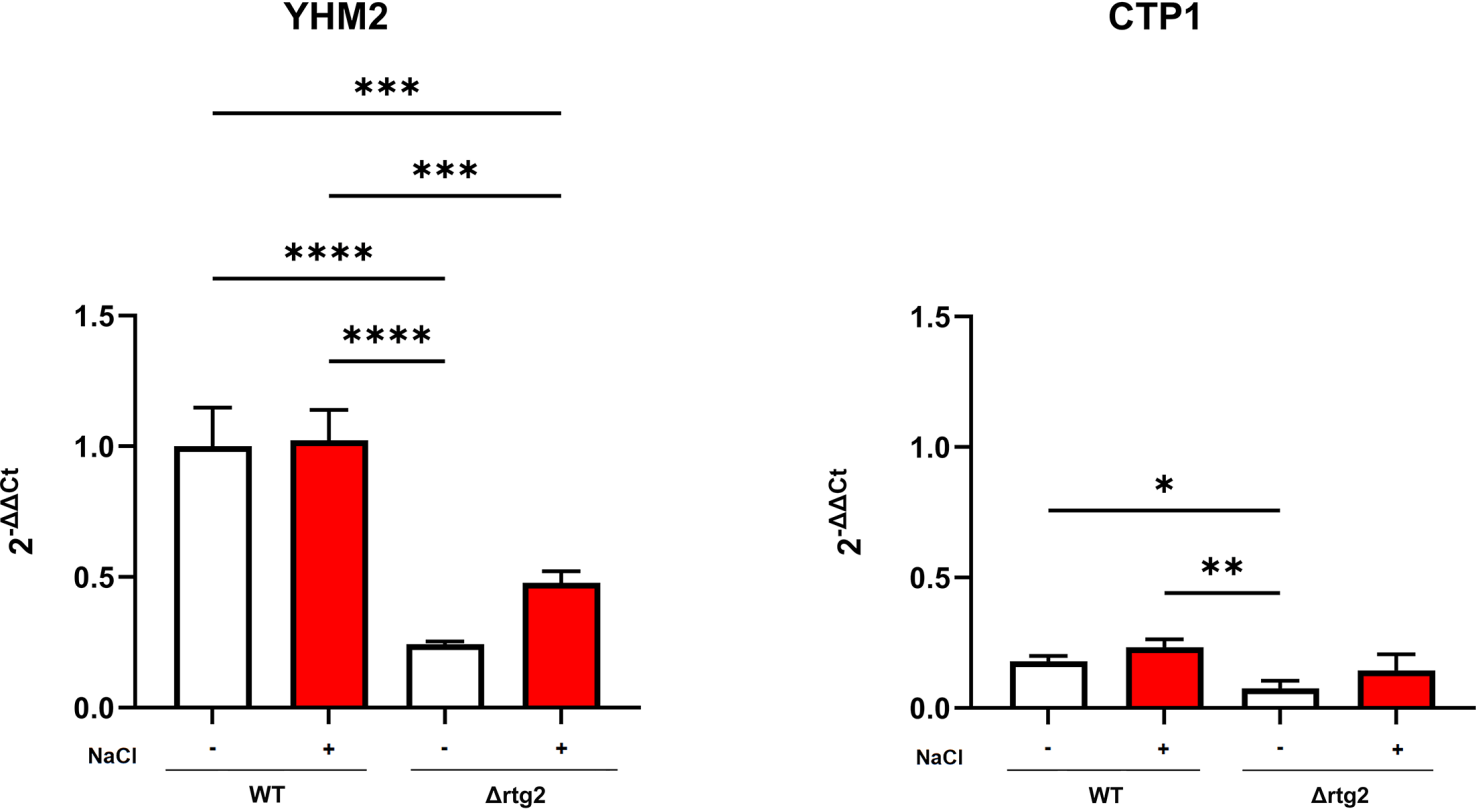
*YHM2* and *CTP1* expression under high-osmotic environment. Wildtype (WT) and mutant cells (Δrtg2) grown overnight in YPD medium were diluted to 0.1 OD_600_ in fresh liquid YPD with (red bars) or without (white bars) 0.8 M NaCl. After 5 h, cells were collected for RNA extraction. Relative amounts of *YHM2* and *CTP1* mRNA were measured by quantitative PCR and calculated according to comparative method (2^−ΔΔCt^), where *YHM2* level in WT cells grown without NaCl stress is used as a calibrator. Values are mean + SD of three independent experiments. One-way ANOVA, Tukey’s (*post hoc*) test; ***P<0.001.

**Figure 5:**
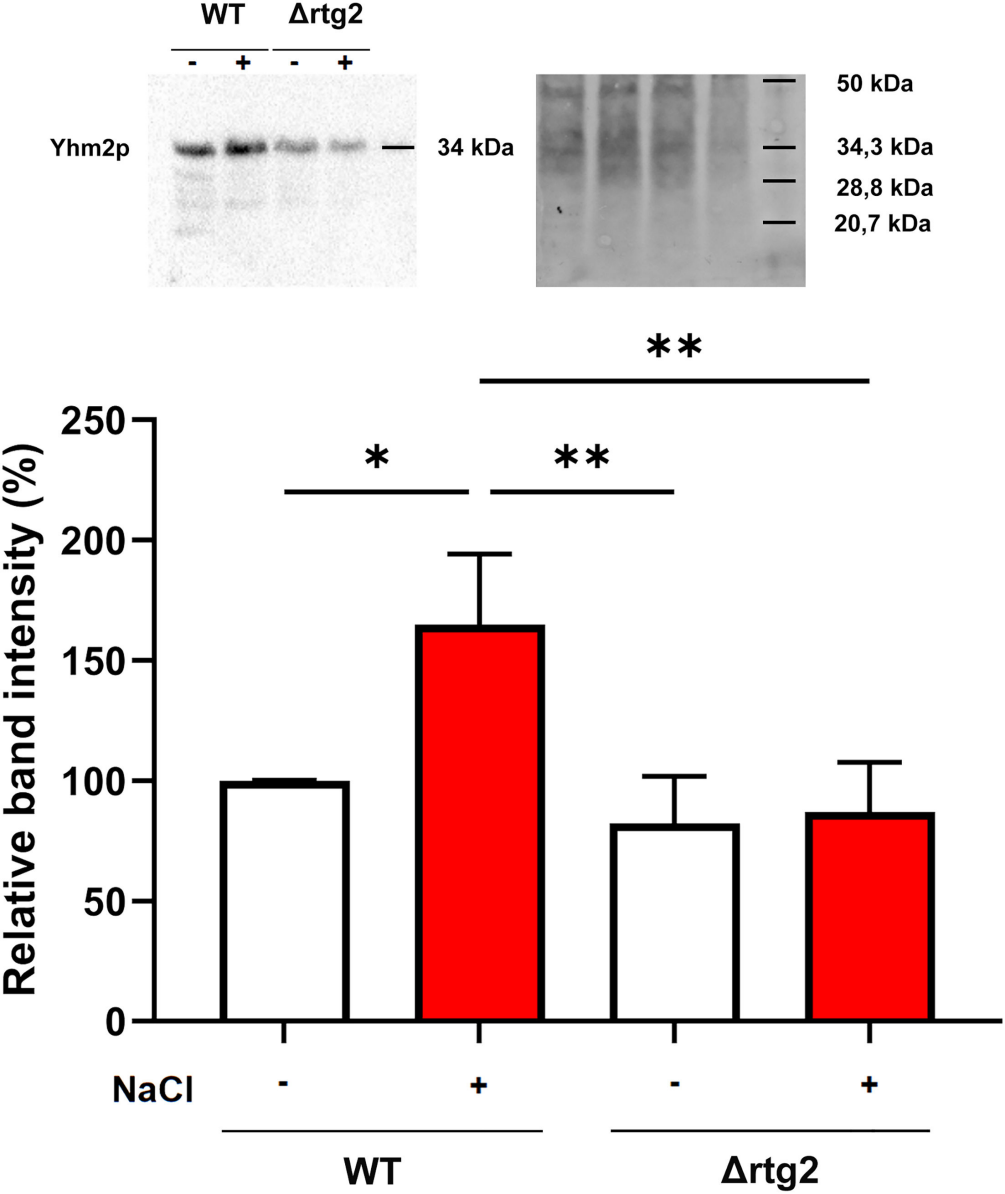
Yhm2p protein levels in WT and Δrtg2 cells under osmotic stress. Western blot analysis of Yhm2p protein in wildtype (WT) and Δrtg2 mitochondria of cells grown in YPD medium for 8 h in the absence or presence of 0.8 M NaCl. Representative western blot images and relative densitometric bar graphs of Yhm2p are shown. Quantification was performed by normalizing band intensities to the total protein signal (Stain-Free) and is expressed as a percentage of amount protein in untreated WT mitochondria. Data are representative of at least three independent experiments.

To gain deeper insight into the role of citrate in the osmoadaptive response of *S. cerevisiae*, we investigated its potential metabolic fate and intracellular distribution under salt stress. As a central metabolic intermediate, citrate might be a substrate for both cytosolic and mitochondrial reactions. Its trafficking between organelles is tightly regulated and relies on specific transporters that ensure proper allocation of citrate to distinct metabolic compartments. Considering the functional cross-talk between peroxisomes and mitochondria, particularly under stress conditions, we focused on the gene expression of the two main mitochondrial citrate transporters, Ctp1p and Yhm2p, in both WT and Δrtg2 mutant cells. *CTP1* encodes for an antiporter, by exchanging citrate from the mitochondrial matrix for another tricarboxylate acid or for malate, while *YHM2* encodes the citrate/2-oxoglutarate carrier involved in the NADPH redox shuttle between mitochondria and cytosol [25–27]. As reported in Figure 4, the expression of both genes was not affected by the presence of NaCl in WT cells. On the other hand, the lack of the *RTG2* gene decreased *YHM2* and *CTP1* expression by about three- and two-fold, respectively, either in the absence or in the presence of stress. In all cases, the level of expression of *YHM2* appeared significantly higher compared with *CTP1,* suggesting a major role for *YHM2* in the osmoadaptive response. Thus, the Yhm2 protein level was assessed in WT and mutant cells with or without NaCl (Figure 5). Western blot analysis showed that NaCl stress causes an increase of Yhm2p in WT cells, while a reduced protein level was observed when comparing WT versus Δrtg2 cells.

**Figure 6:**
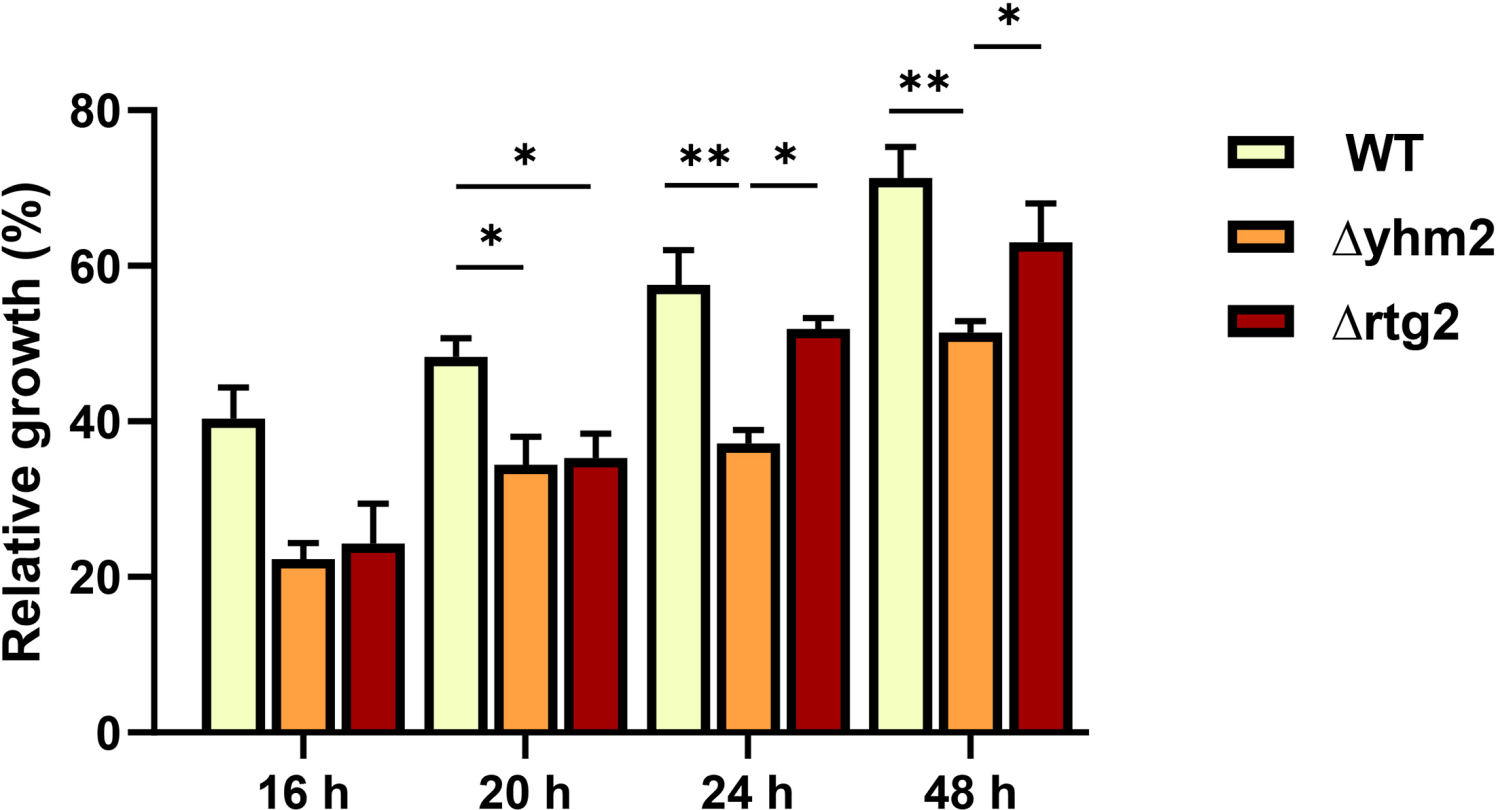
Relative growth of *YHM2* -lacking cells upon osmostress. Wildtype (WT) and indicated mutant cells (Δrtg2, Δyhm2), grown overnight in YPD, were diluted to 0.1 OD_600_ in fresh liquid YPD batch cultures with or without 0.8 M NaCl and optical density (OD_600_) was measured at the indicated times. Relative growth was calculated as the percentage of the OD_600_ of stressed/control cells. Unpaired Student’s *t*-test: a statistically significant difference with *P<0.05; **P<0.005 when comparing WT versus mutant cells from three independent experiments.

To further characterize the role of the citrate/2-oxoglutarate shuttle in osmostress response, the effect of *YHM2* deletion was analyzed on osmosensitivity and compared with WT and Δrtg2 cells. Figure 6 shows cellular relative growth along the time of stress: it is evident that the kinetic response of Δyhm2 cells to NaCl stress is significantly lower compared with WT, while it resembles the phenotype previously observed in Δrtg2 mutants, at least up to 20 h [15,16]. Notably, after 24 h, it becomes even more detrimental.

These data identified Yhm2p as a major player of citrate trafficking in yeast osmoadaptive response.

### Deletion of *YHM2* led to a rewiring in citrate trafficking and metabolism upon osmostress

**Figure 7:**
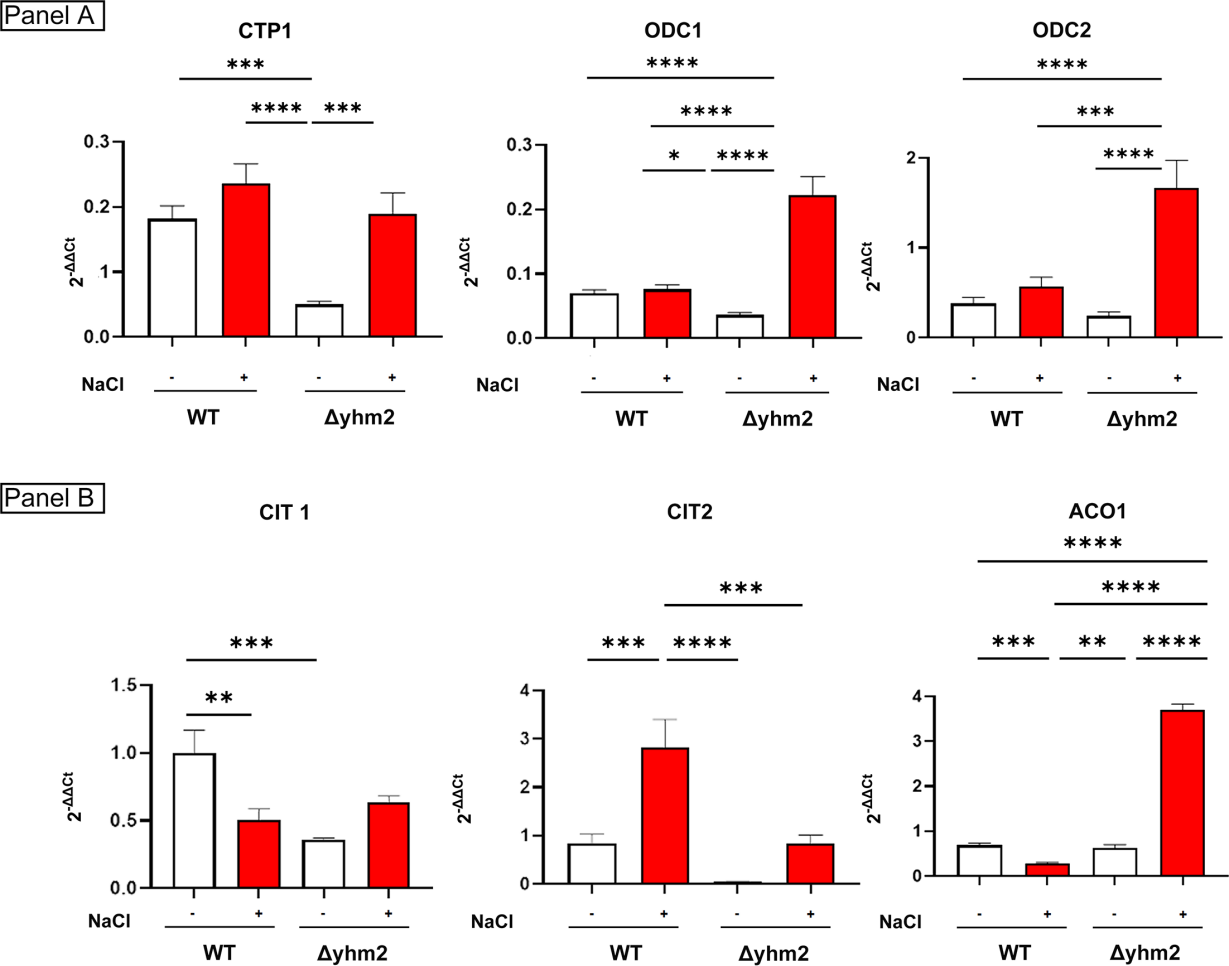
Expression of genes involved in citrate metabolism and transport. Expression of the mitochondrial transporters *CTP1, ODC1,* and *ODC2* (Panel A) and of *CIT1, CIT2, ACO1* (Panel B) was measured in wildtype (WT) and Δyhm2 cells grown in fresh liquid YPD medium with (red bars) or without (white bars) 0.8 M NaCl. After 5 h, cells were harvested for RNA extraction and relative mRNA levels were quantified by qPCR using the comparative method, with *CIT1* expression in WT cells grown without NaCl stress used as the calibrator. Values are mean ± SD of three independent experiments. One-way ANOVA, Tukey’s (*post hoc*) test. ***P<0.001.

Although Yhm2p plays a central role in citrate trafficking and osmoadaptation, its deletion does not lead to lethality, suggesting that compensatory mechanisms may be activated to maintain essential metabolic fluxes. Among the candidate proteins involved in this compensation, there are the mitochondrial carriers Odc1p and Odc2p, previously shown to transport 2-oxoglutarate and related dicarboxylates from mitochondria to the cytosol [29,30]. To investigate whether these transporters contribute to the osmoadaptive response in the absence of Yhm2p, we analyzed the expression levels of *ODC1*, *ODC2*, and *CTP1* in Δyhm2 cells under both control and NaCl stress conditions. Our results indicate that, in the absence of Yhm2p, *CTP1* and *ODC1* expression is significantly reduced under basal conditions, while *ODC2* remains largely unaffected. Upon osmotic stress, all genes are up-regulated: *ODC2* expression is markedly increased, and *CTP1* and *ODC1* levels increase to values comparable with WT cells, suggesting a stress-induced activation of alternative metabolic shuttles. Notably, *ODC2* consistently shows the highest expression among the three carriers under stress, pointing to a potentially prominent compensatory role (Figure 7 panel A).

In parallel, we assessed the expression of genes involved in citrate metabolism. While *CIT1* and *CIT2* are down-regulated in Δyhm2 cells under non-stress conditions, exposure to NaCl results in a moderate recovery of their expression and a strong induction of *ACO1*, encoding aconitase (Figure 7 panel B). These results suggest that the deletion of *YHM2* not only affects the expression of key mitochondrial transporters but also alters the regulation of genes involved in citrate metabolism, particularly in response to osmotic stress.

**Figure 8:**
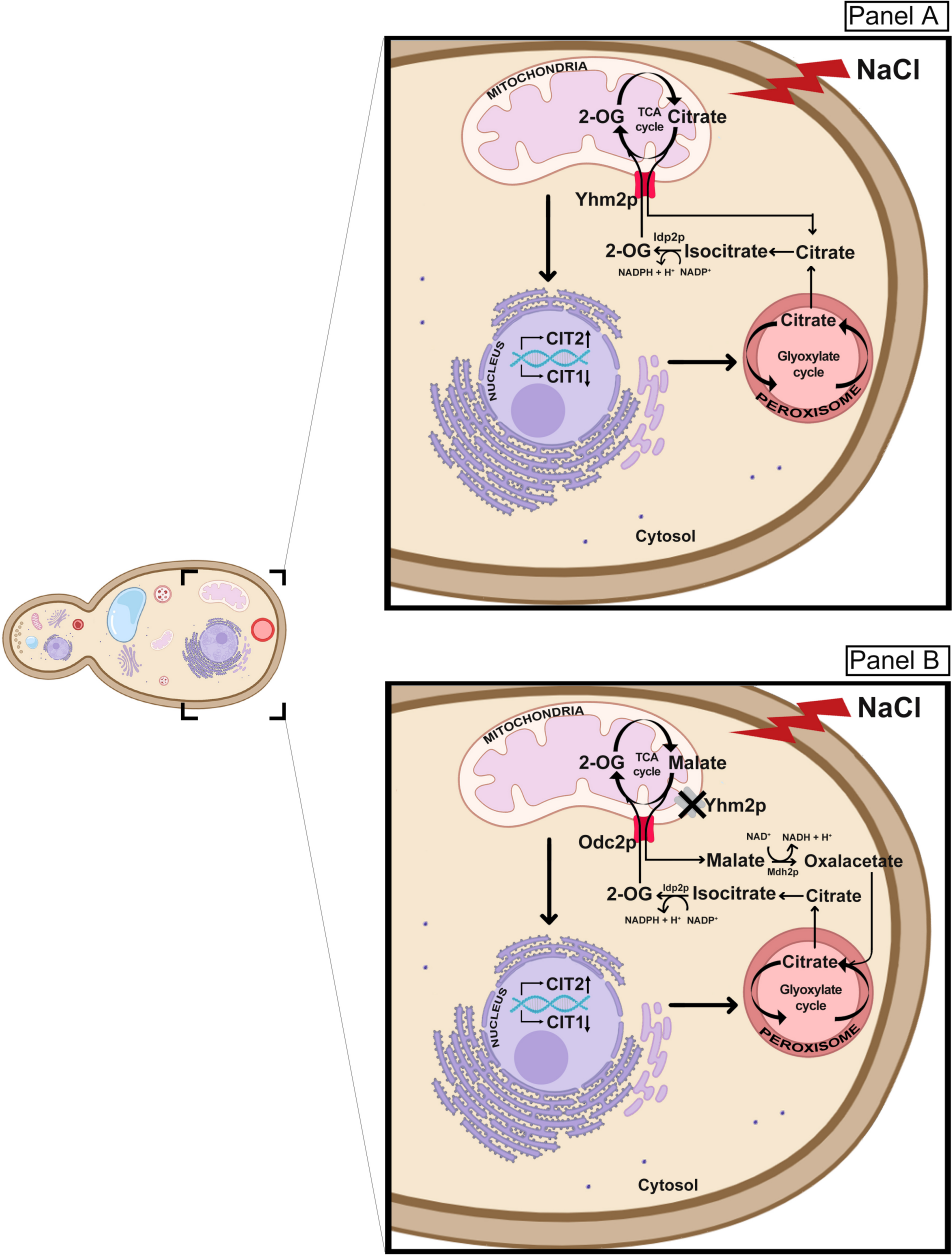
Proposed model for citrate metabolism and trafficking in RTG-dependent osmoadaptive response. **(A**) Under NaCl-induced stress, RTG-dependent citrate synthesis occurs mainly in peroxisomes and cytosol. Cytosolic citrate might be converted to 2-oxoglutarate by the cytosolic NADP-specific isocitrate dehydrogenase Idp2p and then imported into mitochondria via the Yhm2 carrier in exchange with citrate. This would allow the replenishment of TCA cycle with 2-oxoglutarate (**B**) In the absence of *YHM2*, alternative pathways might be activated with the involvement of the mitochondrial transporter Odc2 for metabolites transport (2-oxoglutarate in exchange with malate). Malate might be converted to oxalacetate by cytoplasmic malate dehydrogenase (Mdh2p) and replenish glyoxylate cycle.

### Discussion

Indirect evidence from our previous work suggests that citrate might play a major role in *RTG*-dependent osmoadaptive response of yeast cells [15,16]. In this study, we performed an analysis of TCA cycle metabolites, which revealed a specific increase in citrate levels in comparison with the other TCA cycle intermediates upon NaCl stress (Figure 1). Thus, citrate appears to be the linking metabolite in the inter-organellar cross-talk between peroxisomes and mitochondria. Our data suggest that its synthesis mainly occurs through the peroxisomal isoform of citrate synthase, *CIT2,* while the contribution of the mitochondrial isoforms *CIT1* and *CIT3* is much less relevant (Figure 2). Also, the enzymatic activity of citrate synthase increases upon stress and depends on the RTG pathway, as shown by the significant reduction in cells lacking *RTG2* (Figure 3). Citrate accumulation, along with its reduction in the extracellular medium, may reflect a stress-induced shift toward the TCA cycle, delayed by the down-regulation of aconitase and/or isocitrate dehydrogenase [16]. This delay in the TCA cycle could favor the channeling of citrate toward biosynthetic or protective functions, including NADPH production and redox balancing, while limiting its use in oxidative metabolism. The RTG pathway may facilitate this rewiring, ensuring extramitochondrial, i.e., peroxisomal, citrate production and promoting metabolic plasticity under osmotic stress. Metabolite analysis also revealed a decrease in intracellular succinate (Figure 1A), along with its accumulation in the medium (Figure 1B), suggesting that succinate may be actively exported in the medium under osmotic stress. This export could help relieve redox or metabolic pressure, especially if TCA cycle function is impaired. Similar mechanisms have been reported in mammalian cells under hypoxia or mitochondrial dysfunction, where succinate acts at multiple levels as a signaling molecule for mitochondrial status [31]. This adaptation could be part of an RTG-dependent response to support metabolic flexibility and mitigate oxidative damage under osmostress conditions.

Our data suggest that citrate levels are differentially regulated between peroxisomes/cytoplasm and mitochondria under osmostress, likely reflecting the compartment-specific expression of citrate synthases. Similarly, in mammalian cells, acetyl-CoA/citrate levels can act as messengers from mitochondria to the nucleus and determine cell fate through both metabolic and epigenetic mechanisms [32]. What could be the role of citrate in the inter-organellar cross-talk has been hypothesized by studying the two main mitochondrial carriers involved in the transport of citrate, encoded by *CTP1* and *YHM2*. Although the expression of these two genes needs to be sustained in stressed cells, the significantly higher transcription level of *YHM2* with respect to *CTP1* points to a role of this transporter in the response (Figure 4). Moreover, our data reveal for the first time that the absence of *RTG2* leads to a marked reduction in *YHM2* expression, and this effect appears to be independent of stress conditions (Figure 4). The protein levels of Yhm2 confirmed the dependence on the RTG pathway, especially upon stress conditions (Figure 5). However, the discrepancy observed between *YHM2* mRNA and protein levels (Figures 4 and 5) suggests that its expression could be regulated post-transcriptionally. In this regard, the osmotic stress may enhance Yhm2 protein stability or translation efficiency, independently of transcript abundance [33]. Moreover, the consistently lower Yhm2 protein levels in Δrtg2 cells under both control and stress conditions indicate that *RTG2* may contribute not only to transcriptional regulation but also to the maintenance of Yhm2 protein levels, potentially through indirect effects on mitochondrial function or translational processes.

Yhm2p plays a key physiological role in maintaining cytosolic redox balance by contributing to NADPH generation, which is essential for biosynthetic processes and antioxidant defense. It has been proposed as a component of a mitochondrial–cytosolic citrate/2-oxoglutarate–NADPH shuttle, facilitating the transfer of reducing equivalents [25]. In addition, similar to *CTP1*, deletion of *YHM2* compromises mitochondrial respiratory function by affecting both the structural organization and activity of respiratory complexes [34]. The observed reduction of *YHM2* expression in the absence of *RTG2* and independently on stress conditions (Figure 4) might be part of a co-operative mechanism involving RTG pathway and *YHM2* as a target gene to counteract mitochondrial dysfunction.

Our data provide new insights into the functional relationship between RTG signaling, citrate metabolism, and mitochondrial activity under different cellular demands. Based on these data and on previous evidence, a possible metabolic fate of citrate could be hypothesized: during osmotic stress, RTG-dependent citrate synthesis mainly occurs at the level of peroxisomes and cytoplasm, where citrate can be converted to 2-oxoglutarate through the NADP^+^-dependent reaction catalyzed by the cytosolic isocitrate dehydrogenase (Idp2p). 2-Oxoglutarate, in turn, may be transported into the mitochondria in exchange with mitochondrial citrate, synthesized by Cit1p, through the Yhm2 carrier. This bidirectional exchange ensures a continuous supply of 2-oxoglutarate to the TCA cycle, supporting mitochondrial metabolism under stress (Figure 8 panel A). However, this citrate/2-oxoglutarate exchange also plays a critical role in cytosolic NADPH production. The metabolomic data support this model, since only succinate and fumarate, intermediates downstream of 2-oxoglutarate, accumulate in the medium of osmotically stressed cells (Figure 1B), indicating sustained TCA cycle activity beyond this point.

The importance of Yhm2p, particularly in the long-term response to osmotic stress, is underscored by the growth defect observed in Δyhm2 cells under NaCl stress (Figure 6).

In the absence of Yhm2p, we propose that alternative adaptive mechanisms could be activated with the involvement of other mitochondrial carriers. Odc2p, which exchanges cytosolic 2-oxoglutarate with mitochondrial malate (and, to a lesser extent, oxaloacetate), may partially compensate for citrate transport. Once malate is exported to the cytosol and converted into oxaloacetate, it may contribute to citrate synthesis via Cit2p, thereby feeding into the NADPH-producing Idp2p reaction and closing the metabolic loop (Figure 8 panel B). Furthermore, in the absence of *YHM2*, cells activate compensatory transcriptional programs to preserve metabolic homeostasis during osmotic stress. Notably, the observed up-regulation of *CIT1* and *ACO1* in Δyhm2 cells exposed to NaCl may reflect an adaptive attempt to reinforce mitochondrial citrate production and sustain TCA cycle activity, despite impaired citrate export. In this context, increased *CIT1* expression may serve to boost intramitochondrial citrate synthesis, ensuring substrate availability for energy production and biosynthetic reactions. Concurrently, enhanced *ACO1* expression could facilitate the conversion of citrate to isocitrate, supporting continued flux through the TCA cycle and downstream production of 2-oxoglutarate.

The compartmentalization of metabolic processes between peroxisomes and mitochondria under osmotic stress could reduce the burden on each organelle, optimize resource use, and mitigate ROS accumulation. The division of functions raises an important question about the nature of the metabolic communication between organelles. It is still unclear whether the metabolic communication between mitochondria and other organelles is unidirectional or bidirectional. The directionality of this signaling remains speculative, as mitochondria could act both as receivers and transmitters of stress cues. One possible scenario is that NaCl stress primarily affects mitochondrial function, thereby activating the RTG retrograde signaling through transcriptional regulation in the nucleus. Alternatively, changes in citrate levels—either in the cytoplasm or within mitochondria—might themselves serve as triggering signals for nuclear transcriptional activation, subsequently co-ordinating the communication between peroxisomes and mitochondria. The mechanisms and messengers responsible for transmitting information from one organelle to the other remain largely unclear. Nonetheless, possible communication strategies might involve (i) release of biomolecules—such as proteins, ROS, or metabolites—that influence the function of the other organelle either directly or indirectly (for instance, via epigenetic changes), or (ii) triggering membrane-associated signaling scaffolds that regulate the activity of both organelles [21].

In summary, our study underscores the critical role of the RTG pathway in orchestrating citrate biosynthesis and trafficking as part of a broader metabolic adaptation to osmotic stress. The co-ordinated regulation of *CIT1* and *CIT2*, along with the modulation of citrate carrier, reveals citrate as a central component in the peroxisome–mitochondria-nucleus axis in maintaining metabolic homeostasis. Given the centrality of citrate in redox balance and energy metabolism, similar mechanisms could operate in higher eukaryotes, with potential implications for biomedical research and therapeutic development. Indeed, the human mitochondrial SLC25A1 protein transports citrate between mitochondria and cytosol, thereby regulating citrate levels within the cell and significantly influencing acetyl-CoA and pyruvate availability [35].

In conclusion, our findings uncover a previously uncharacterized metabolic axis that links peroxisomal citrate production to mitochondrial function through a citrate shuttling mechanism during osmotic stress. This study demonstrates for the first time that the RTG signaling modulates mitochondrial adaptation through the control of the Yhm2-mediated citrate/2-oxoglutarate shuttle. This mechanism would ensure the supply of 2-oxoglutarate to sustain mitochondrial metabolism, redox balance, and long-term survival under stress conditions. The identification of citrate and 2-oxoglutarate as signaling and metabolic intermediates to connect organelle functions highlights a novel layer of inter-organelle communication, expanding the functional landscape of retrograde signaling. These insights not only deepen our understanding of metabolic rewiring under stress in yeast but may also have broader relevance for conserved stress response mechanisms in higher eukaryotes.

## Materials and methods

### Yeast strains and growth conditions

The *S. cerevisiae* strains used in this study were W303-1B (MATα *ade2 leu2 his3 trp1 ura3*) and derivatives W303-1B ∆rtg2 and ∆yhm2. The deletion strains were constructed using the PCR-mediated gene disruption technique by replacing the open reading frame of the genes with LEU2 [8] or KanMX3 [36] for Drtg2 and ∆yhm2, respectively. All deletions were verified by PCR.

Cells were grown aerobically at 30°C in YPD (1% yeast extract, 2% bactopeptone (GIBCO, Life Technologies, Waltham, MA, U.S.A.), and 2% D (+) glucose monohydrate (ROTH) with 2% agar (Biokar diagnostics, France) for solid medium in the absence or in the presence of 0.8 M sodium chloride (NaCl). For batch-culture growth assays, fresh yeast cells cultured for about 16 h at 30°C were diluted in flasks to the initial OD600 of 0.1 and optical density was monitored at 600 nm by using a Thermo Spec-tronic Genesis 20 spectrophotometer. Relative growth was calculated as the percentage of the optical density values under stress conditions (with NaCl) compared with the control (without NaCl) at different times. At least three independent cultures were analyzed for each condition in each independent experiment.

### Metabolomic analysis (UPLC-MS/MS)

Yeast cells grown at 30°C in YPD in the presence and in the absence of NaCl for 8 hours were collected and disrupted using a methanol (LC-MS grade) to ultrapure water solution at an 80:20 ratio in order to quantify intracellular compounds. For the quantification of extracellular compounds, 100 µl of the growth medium was collected and subsequently extracted with pure methanol (LC-MS grade). Following the evaporation of the solvent, the resulting pellets were reconstituted in a mixture of acetonitrile (LC-MS grade) and ultrapure water (40:60). The samples were then analyzed via ultra performance liquid chromatography-tandem mass spectrometry (UPLC-MS/MS) using an Acquity UPLC system (Waters) equipped with a HSS T3 Column (100 Å, 1.8 µm, 2.1 mm × 100 mm, Waters). The system operated at a flow rate of 0.3 ml/min and was coupled to a Quattro Premier mass spectrometer. Detailed multiple reaction monitoring (MRM) transitions employed in the analysis are available in Table 1.

**Table 1:** MRM transitions used for metabolite detection

Metabolites	Transition	Ion mode
Isocitrate	191.1 > 155,0	Negative
Citrate	191.1 > 110.9	Negative
2-oxoglutarate	145.1 > 101.1	Negative
Malate	133,0 > 115.0	Negative
Succinate	117.05 > 73,0	Negative
Fumarate	115.1 > 70.9	Negative

### Quantitative PCR (qPCR)

The mRNA levels of investigated genes were determined in continuously growing cells after 5 h of NaCl exposure and in the absence of stress. A total of 5 × 10^7^ cells were collected and centrifuged at 3000 g and stored at −80°C before total RNA extraction with a Presto Mini RNA Yeast Kit (Geneaid, New Taipei City, Taiwan). After RNA extraction, we immediately performed the cDNA synthesis preceded by DNase treatment, using Superscript IV VILO Mastre Mix (Invitrogen, Thermo Fisher Scientific, Waltham, MA, U.S.A.) according to the manufacturer’s instructions. cDNA was directly used for the quantitative PCR (qPCR) analysis or stored at −20°C. Quantitative PCR experiments were carried out using the QuantStudio 3 Real-Time PCR System (Applied Biosystems, Thermo Fisher Scientific, Waltham, MA, U.S.A.) and the primer pairs (Table 2) designed with PrimerExpress3.0 (AppliedBiosystems, ThermoFisher Scientific, Waltham, MA, U.S.A.) and purchased from Invitrogen (Thermo Fisher Scientific, Waltham, MA, U.S.A.). The qPCR reactions were assembled to a final volume of 20 µl containing 20 ng of reverse-transcribed first-strand cDNA, 10 µl of SYBR Select Master Mix (Applied Biosystems, Thermo Fisher Scientific, Waltham, MA, U.S.A.) and 300 nM of each primer. The specificity of the PCR amplification was checked with the heat dissociation protocol after the final cycle of PCR.

**Table 2:** Primers used for quantitative PCR.

Gene	Primer	Sequence
ACO1	Forward	5′- CAAGAACCCAGCTGACTATGACA-3′
ACO1	Reverse	5′- CCAATTCAGCTAGACCCAGAATATC-3′
CIT1	Forward	5′-GCGCCTCCGAACAAACG-3′
CIT1	Reverse	5′-CTGCCTTTGCTGGGATAATTTC-3′
CIT2	Forward	5′-TGTAAGGCAATTCGTTAAAGAGCAT-3′
CIT2	Reverse	5′-CCCATACGCTCCCTGGAATAC-3′
CIT3	Forward	5′-CCCTGCTCGGCATGACA-3′
CIT3	Reverse	5′-GCTGGGAAGTAAGGTTGCATGT-3′
YHM2	Forward	5′-CTCGTTTTGGTTTGTCCAGACTAG-3′
YHM2	Reverse	5′-TTCTCGAACGGATTCAACTTGTC-3′
CTP1	Forward	5′-CATAATAATGGGCGCGGTGTA-3′
CTP1	Reverse	5′-GTTTGCGGCCTGTCTCATG-3′
ODC1	Forward	5′-CATCCCGCTGTTGTTGCA-3′
ODC1	Reverse	5′-TCCATCACGCCCGTGTAGT-3′
ODC2	Forward	5′-GACCATGCTTAACACTCCCTTTG-3′
ODC2	Reverse	5′-CGCGTCCACACTTTGGATT-3′
ACT1	Forward	5′-ACTTTCAACGTTCCAGCCTTCT-3′
ACT1	Reverse	5′-ACACCATCACCGGAATCCAA-3′

To correct for differences in the amount of starting first-strand cDNAs, the *ACT1* mRNA was amplified in parallel as a reference gene. The relative quantification of the investigated genes was performed according to the comparative method (2^−∆∆Ct^) [37] whereby the fold change of the investigated genes was calculated relative to the WT cells that were grown without NaCl stress and used as the calibrator.

### Citrate synthase activity assay

Yeast cells, grown at 30°C for 8 h in YPD in the presence and in the absence of NaCl, were washed and then broken by vigorous shaking with an equal volume of glass beads in a buffer containing 50 mM Tris-HCl pH 7.4 and a mixture of protease inhibitors. Centrifugations (700 g, 2 min) allowed the elimination of unbroken cells and glass beads. Cellular proteins were quantified by the Bradford method. For citrate synthase assay, 1 ml of reaction mixture was prepared that contained 75 mM Tris -HCl buffer (pH 8.0), 0.1% Tx-20, 0.5 mM oxaloacetate, 0.1 mM DTNB, and saturating amounts of acetyl-CoA substrate (0.4 mM). Citrate synthase (2.3.3.1) activity was determined by monitoring the oxidation of coenzyme A by 5,5′-dithiobis-2-nitrobenzoic acid (DTNB) as a function of time at 412 nm, in a Cary 50 SCAN UV-Visible spectrophotometer (Varian). The enzyme activity was calculated using an extinction coefficient of 13, 6 M^−1^cm^−1^. One citrate synthase unit was equal to 1 μmole of DTNB reduced per minute per mg dry weight.

### Preparation of mitochondria and western blot analysis

For the isolation of mitochondria, yeast cells were grown at 30°C in YPD medium for 8 hours in the absence and in the presence of NaCl. Organelles from yeast lysates were harvested according to standard procedures [38] with appropriate modifications. Yeast cells were collected by centrifugation (5 min at 4000 rpm), washed once with distilled water, suspended to 0.5 g /ml in 0.1 M Tris-SO_4_ pH 9.4, 10 mM dithiothreitol, and incubated for 10 min at 30°C. They were then washed once with 1.2 M sorbitol and suspended in 1.2 M sorbitol, 20 mM KPi, pH 7.4, to give 0.15 g of cell, wet weight/ml. Zymolyase 5000 (5 mg/g of cell, wet weight) was added, and the suspension was incubated at 30°C with gentle shaking. Conversion to spheroplasts was checked with a spectrophotometer at 578 nm. After 50–60 min, all the cells had usually been converted to spheroplasts. From this point on, all operations were carried out at 0-4 °C. Spheroplasts were harvested by centrifugation for 5 min at 4000 rpm, washed with 1.2 M sorbitol and harvested by centrifugation for 5 min at 4000 rpm again. For homogenization, spheroplasts were suspended in 0.6 M sorbitol, 10 mM TrisC1, pH 7.4, 1 mM EDTA, 0.1% bovine serum albumin, 1 mM phenylmethylsulfonyl fluoride to a concentration of 0.08 g of spheroplasts (wet weight)/ml. After chilling on ice, spheroplasts were homogenized by 10–15 strokes in a glass potter. The homogenate was diluted with 1 vol of the homogenization buffer and centrifuged for 5 min at 3000 rpm. The supernatant was saved and centrifuged at 14000 rpm for 10 min. The mitochondrial pellet was resuspended in STE (0.25 M sorbitol, 20 mM Tris-Cl, EDTA 1 mM pH 7.4) and was re-homogenized as before and recentrifuged at 3500 rpm. The supernatant was saved and centrifuged at 15000 rpm for 20 min. The resulting mitochondrial fraction was then resuspended in STE and quantified by Bradford assay using bovine serum albumin as standard. 10 μg of mitochondrial fraction for each strain was separated by 10% SDS-PAGE. Equal loading was confirmed by imaging the Stain-Free gel prior to transfer. After electro-transfer onto nitrocellulose membrane (Biorad), the membrane was incubated with rabbit antiserum against the bacterially expressed Yhm2p protein [25] and detection was performed using HRP-conjugated secondary antibodies and enhanced chemiluminescence (ECL) reagents, according to the manufacturer instructions. Total protein normalization was performed using Stain-Free technology (Biorad) and signal quantifications were done using the Image Lab software (2020, BIORAD laboratories).

### Statistical analysis

All the experiments were repeated at least three times, and the results are reported as means with standard deviation. Student’s t-test was used to determine significant differences between samples, using Microsoft Excel software (Microsoft® Excel® per Microsoft 365 MSO, Versione 2405 Build 16.0.17628.20006) with significant differences reported as*P* values between 0.05 and 0.0001 for all results. For UPLC-MS/MS and qPCR data, one-way ANOVA followed by Tukey’s multiple comparison test was used.

## Data Availability

Raw data from this study are available upon reasonable request from the corresponding author.

## Open Access

This article has been published open access under our Subscribe to Open programme, made possible through the support of our subscribing institutions, learn more here: https://portlandpress.com/pages/open_access_options_and_prices#conditional

## Abbreviations

MRM: multiple reaction monitoring
2-OG: 2-oxoglutarate
ROS: reactive oxygen species
qPCR: quantitative PCR
